# Development and Potential Application of Ras Domain Containing Protein from *Haemonchus contortus* for Diagnosis of Goat Infection

**DOI:** 10.3390/ani10010138

**Published:** 2020-01-15

**Authors:** Kalibixiati Aimulajiang, Man Cao, Shuyi Liao, Muhammad Ali-ul-Husnain Naqvi, Xiaowei Tian, Zehua Li, Mingmin Lu, Shakeel Ahmed Lakho, Xiangrui Li, Lixin Xu, Xiaokai Song, RuoFeng Yan

**Affiliations:** Ministry of Education (MOE) Joint International Research Laboratory of Animal Health and Food Safety, College of Veterinary Medicine, Nanjing Agricultural University, Nanjing 210095, China; 2017207022@njau.edu.cn (K.A.); 2018107075@njau.edu.cn (M.C.); 2017107077@njau.edu.cn (S.L.); 2017207047@njau.edu.cn (M.A.-u.-H.N.); 2017207011@njau.edu.cn (X.T.); 2017807168@njau.edu.cn (Z.L.); 2015207018@njau.edu.cn (M.L.); 2017207046@njau.edu.cn (S.A.L.); lixiangrui@njau.edu.cn (X.L.); xulixin@njau.edu.cn (L.X.); songxiaokai@njau.edu.cn (X.S.)

**Keywords:** *Haemonchus contortus*, Ras domain-containing protein, serological diagnosis, Western blotting, indirect ELISA

## Abstract

**Simple Summary:**

The early diagnosis of Haemonchosis is important for the control and effective treatment of the disease. Early stage detection of *Haemonchus contortus* infection has not been attempted in goats, even though the immature worms are blood-sucking. This study was carried out to detect *H. contortus* infection during the early and late stages in goats. The results of this study assessed that recombinant *H. contortus* Ras domain-containing protein (rHcRas) could detect the antibody in *H. contortus*-infected goat sera during the prepatent and postpatent periods with high sensitivity and specificity using immunodiagnostic techniques. Our findings illustrated that indirect ELISA based on rHcRas also had the potential to detect *H. contortus* infection from field samples.

**Abstract:**

*Haemonchus contortus* is an important gastrointestinal nematode of small ruminants that causes significant mortality in goats worldwide. Diagnosis of this infection mainly depends on the evaluation of clinical signs and fecal examination. However, limitations often occur in early or mild infections. For this purpose, serological diagnosis seems to be more accurate and reliable. Ras domain-containing protein (Ras) is one of *H. contortus*’s excretory and secretory products (ESPs) that can be isolated from different larval stages of the nematode. In this study, the recombinant *H. contortus* Ras domain-containing protein (rHcRas) was expressed and purified and its diagnostic potential was evaluated. Reactions between rHcRas and goat sera were tested using Western blotting (WB). The results showed that rHcRas could be recognized by sera as early as 14 days post infection (DPI), and antibodies against rHcRas in infected goats could be maintained for over 89 days. No reaction was found between rHcRas and antibodies against *Trichinella spiralis*, *Fasciola hepatica,* or *Toxoplasma gondii*. An indirect enzyme-linked immunosorbent assay (ELISA) was produced based on rHcRas. The optimal coating antigen (157 ng of rHcRas/well) and serum dilutions (1:50) were determined via checkerboard titration. Indirect ELISA based on rHcRas showed 87.5% sensitivity and 90.6% specificity. The cut-off values for this experiment were determined to be 0.324 (positive) and 0.273 (negative), respectively, and the variation coefficient (CV) was less than 15%. The results of the indirect ELISA in-field examination showed that 17.6% (9/51) of the goats were infected with *H. contortus*, higher than the fecal examination results (15.7%, 8/51). When compared the results of the indirect ELISA and necropsy testing, 98.0% (50/51) consistency was found. These results indicated that rHcRas was a potential antigen for the diagnosis of *H. contortus* infection in goats.

## 1. Introduction

*Haemonchus contortus* is a highly pathogenic, blood-sucking parasite that causes huge production losses of goats worldwide [1]. Young animals are more susceptible to Haemonchosis compared to adults [2]. The diagnosis of Haemonchosis is usually based on the detection of clinical signs such as severe anemia. Large numbers of *H. contortus* in the abomasum verify the presence of the parasite postmortem [1]. However, clinical signs appear in the late stage of infection. In addition, parasite eggs can be detected in feces after the prepatent period (21–28 days), although identification of parasitic stages in feces requires trained technical personnel [3,4]. Molecular diagnostic tests, i.e., loop-mediated isothermal amplification and quantitative polymerase chain reaction, have major significance in enhancing the diagnostic capabilities of Haemonchosis. Nevertheless, their applications are limited to the laboratory, as more sophisticated equipment is required [4,5]. Thus, the development of a convenient, rapid, and profitable diagnostic method is needed, especially for the detection of clinical samples.

The early diagnosis of Haemonchosis is important for the control and effective treatment of the disease. In this context, the detection of antibodies against *H. contortus* antigens via immunological methods is a useful alternative for early and rapid diagnosis. In addition, these techniques are dependent on antigen detection, which is inexpensive and easy to implement. Earlier were conducted to evaluate the diagnostic potential of different parasite recombinant peptides through these techniques [6,7,8,9].

Excretory and secretory products (ESPs) play a major role in *H. contortus* infection, consisting of several proteins which can modulate or suppress the host’s immune response. Ras domain-containing protein (HcRas) is a Haemonchus ESP which binds to goat Peripheral blood mononuclear cells, which are produced during different developmental stages (7th, 15th, 40th, and 60th days) in vivo [10]. However, its diagnostic potential is still unknown. Ras-related proteins are crucial regulators of intracellular vesicular transporters, which are potential candidates for immunological applications [11]. In this study, Ras was purified and expressed, and antibodies were detected at different levels of *H. contortus* infection and evaluated via immunoblotting. Indirect ELISA was established and optimized based on the HcRas antigen. The diagnostic potential of HcRas was also evaluated using sera samples collected from the field.

## 2. Materials and Methods

### 2.1. Expression of Recombinant Proteins

Recombinant plasmid expression HcRas (pET(plasmid *E. coli* T7)-28a (+)/HcRas; Uniprot: U6NXR0) was provided by MOE joint international Research Laboratory of Animal Health and Food Safety, College of Veterinary Medicine, Nanjing Agriculture University. Recombinant plasmids (HcRas) were transformed into *Escherichia coli* BL21-competent cells (Vazyme Biotech, Nanjing, Jiangsu, China) and cultured in Luria Bertini (LB) medium containing ampicillin at 100 µg/mL (AMP). The positive colonies were grown in LB broth at 37 °C with constant shaking at 120 rpm until an optical density at 600 nm reached 0.6–0.8; the recombinant protein was then induced with 1 mM isopropyl β-D-1-thiogalactopyranoside (IPTG; Sigma Aldrich, USA) for 5 h. Afterward, the cultures were harvested and centrifuged at 10,000× *g* for 10 min. The pellets were lysed with lysozyme (10 µg/mL; Sigma-Aldrich), followed by sonication. The sonication products of the recombinant proteins were examined with a 12% SDS-PAGE. Histidine (His)-tagged fusion proteins were purified from the bacterial lysates using Ni^2+^ nitrilotriacetic acid column (GE Healthcare, Pittsburgh, PA, USA), according to the manufacturer’s instructions. Finally, recombinant protein production was analyzed with an SDS-PAGE and the concentration was determined by Bradford [12] and stored at −80 °C.

### 2.2. Animals and Parasites

Five crossbred female goats (body weight, 30.6 ± 2.7 kg) at 4–6 months old were bought from Xuyi County, Jiangsu Province, China. The goats were kept in the animal house at Nanjing Agricultural University and provided with whole-shelled corn, hay, and water ad libitum. The goats were confirmed as helminth-free after fecal examination for a period of 4 weeks.

*H. contortus* L3 (third-stage) larvae were maintained by the MOE joint international Research Laboratory of Animal Health and Food Safety, College of Veterinary Medicine, Nanjing Agriculture University, for use in this experiment.

### 2.3. Serum Sample

Five crossbred goats were experimentally infected with 8000 *H. contortus* L3. Serum samples were collected from each goat before infection (negative control) and at 7, 14, 21, 35, 49, 63, 85, and 103 DPI.

Another 32 positive serum samples from *H. contortus*-infected goats and 32 negative serum samples from non-infected goats were collected from another independent experiment. These serum samples were used to evaluate the sensitivity, specificity, and cut-off value of the ELISA.

Serum samples infected with *Fasciola hepatica* were kindly provided by Professor Huang Siyang, Yangzhou University. Serum samples infected with *Toxoplasma gondii* and *Trichinella spiralis* were provided by MOE joint international Research Laboratory of Animal Health and Food Safety, College of Veterinary Medicine, Nanjing Agriculture University.

### 2.4. Immunoblot Analysis of Recombinant Antigens

The recombinant proteins were separated on 12% SDS-PAGE and subsequently transferred to a 0.22-μM-pore nitrocellulose filter membrane (NC, Merck Millipore, Tullagreen, Carrigtwohill, Ireland) using a semi-dry system (Bio-Rad, Hercules, CA, USA). The transferred membrane was blocked with 5% skim milk (BD, Baltimore, MD, USA) diluted in TBS (Tris buffered saline) with 0.5% Tween-20 (TBST) for 2 h at 37 ℃ and incubated with goat serum diluted by 1:100 in blocking buffer for 1 h at 37 °C. After 3 washes with TBST, the strips were probed with a 1:4000 diluted secondary antibody, horseradish peroxidase (HRP)-conjugated rabbit anti-goat IgG (Thermo Fischer Scientific, Waltham, MA, USA). Then, the strips were washed with TBST five times and the immunoreactions were evaluated using High-sig Electro-Chemi-Luminescence (ECL, Thermo Fischer Scientific, Waltham, MA, USA) Western blotting substrate.

### 2.5. Indirect ELISA Procedure

An indirect ELISA was performed to assess the immunodiagnostic potential of rHcRas. Checkerboard titrations were used to optimize the best working dilution of the rHcRas (39–628 ng/well) and the serum samples (1:50 to 1:200 dilutions). The incubation time (30, 45, 60, and 90 min) for the serum (primary antibody) and the rabbit anti-goat IgG-HRP (second antibody) reactions and the secondary antibody concentration were also optimized. The optical density was measured at 450 nm (OD450) with a Microplate reader (Thermo Fischer Scientific, Waltham, MA, USA). The highest specific value between the positive and negative samples was picked as a standard for the subsequent experiments.

Confirmation of optimal conditions was performed by an indirect ELISA as described previously [6]. The optimum concentration of diluted antigen with 100 μL of coating buffer (0.05 M carbonate buffer, pH 9.6) was incubated at 4 °C overnight in 96-well ELISA plates (Costar, Bodenheim, Germany). After three washes with TBST, the plates were blocked with 5% BSA (Bovine Serum Albumin) for 60 min at 37 °C, followed by three washes with TBST. Positive and negative goat sera of 100 μL each diluted in blocking buffer were added to the wells in triplicates followed by incubation for 90 min at 37 °C. After washing with TBST three times, rabbit anti-goat IgG conjugated with HRP (Thermo Fischer Scientific, Waltham, MA, USA) was added and incubated for 45 min at 37 °C. Subsequently, five washes with TBST were performed and the peroxidase reaction was visualized by using 100 μL/well ready-to-use tetramethylbenzidine-hydrogen peroxide (TMB) solution as a substrate for 10 min at room temperature and stopped by adding 100 μL/well 0.5 M H_2_SO_4_. The optical density (OD) values of the wells were detected at an absorbance of 450 nm using a Microplate reader (Thermo Fischer Scientific, Waltham, MA, USA).

### 2.6. Estimation of Cut-off Value, Sensitivity, and Specificity

The negative cut-off value was determined by taking the mean OD450 of the negative samples + 2 multiplied by the standard deviation (SD), and the positive cut-off value by taking the mean OD450 of the positive samples + 3 multiplied by SD [13,14]. The intervals between the negative and positive cut-off values were considered to be false negative/positives.

To investigate the further feasibility of the indirect ELISA, positive serum samples (n = 32) from goats with *H. contortus* and serum samples (*n* = 32) from non-infected goats were evaluated to calculate the sensitivity and specificity by applying the formulae described previously [15]:

(1) $$\mathrm{Sensitivity}=\frac{\mathrm{Positive}}{\mathrm{Positive}+\mathrm{False}\;\mathrm{Negative}},$$

(2) $$\mathrm{Specificity}=\frac{\mathrm{Negative}}{\mathrm{Negative}+\mathrm{False}\;\mathrm{Positive}}.$$

In addition, 9 serum samples positive for *T. spiralis* (*n* = 4), *F. hepatica* (*n* = 1) and *T. gondii* (*n* = 4) were used to estimate cross-reactivity.

### 2.7. Measurement of Repeatability

Six positive (P) and six negative (N) goat serum samples were used to test the repeatability of the rHcRas-ELISA. Three positive and three negative sera samples were collected from different goats at different time points and an indirect ELISA was performed within the run in triplicate. Between the runs, three plates were operated and repeated on 3 different occasions. The repeatability was expressed through a coefficient of variation (CV) percentage, i.e., the standard deviation (SD) expressed as a percentage over the mean (x) of the repeats [16]:

(3) $$\mathrm{CV}\;=\;\frac{\mathrm{SD}}{\bar{x}}\;\times 100\%$$

### 2.8. Field Evaluation of the Indirect ELISA

To evaluate the indirect ELISA in regard to the field examinations, 51 goats were randomly selected and bought from different goat farms in Nanjing, Jiangsu Province, China, to collect the serum samples; *H. contortus* infection was detected by the ELISA constructed in this study. To compare the results with fecal examination, fecal samples were collected in parallel and tested via a conventional fecal egg count (FEC), as described previously [17]. Briefly, fecal samples were collected directly from the anus by inserting the middle finger, and the samples were kept in labeled plastic bags until use. Then, 2 g of feces were dispersed in 58 mL of saturated NaCl and both chambers of the McMaster slide were filled with this solution using a transfer pipette. The slide was kept still for 5 min to allow parasitic eggs to float on the surface; all eggs inside the grid areas were counted under a microscope using 10 × magnification. One chamber was examined under the microscope and the number of eggs under one area was multiplied by 200, or two chambers were examined and multiplied by 100, to calculate the total number of eggs per gram (EPG) of the feces [18]. Goats positive for *H. contortus* infection were slaughtered humanely to check for the presence of worms in the abomasum and the number of worms was counted. The treatments of animals in our research conformed to the guidelines of the Animal Ethics Committee, Nanjing Agricultural University, China. The protocols of our experiments were all approved by the Science and Technology Agency of Jiangsu Province. The approval ID was SYXK (SU) 2010–0005.

## 3. Results

### 3.1. Expression, Purification, and Western Blot Analysis of rHcRas

**Figure 1:**
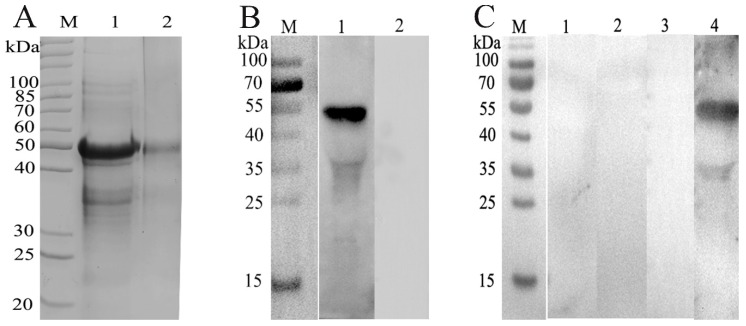
(**A)** SDS-PAGE. Lane M: Standard protein molecular weight marker; lane 1: rHcRas (recombinant *H. contortus* Ras domain-containing protein) was expressed after induction with IPTG (isopropyl β-D-1-thiogalactopyranoside); lane 2: purified rHcRas. (**B**) Western blot. Lane 1: rHcRas was recognized by sera from goats infected with *H. contortus*; lane 2: no reactions between rHcRas and normal goat serum (negative control). (**C**) Western blot to evaluate the specificity of rHcRas. Lane 1: no reaction with antibody against *F. hepatica*; lane 2: no reaction with antibody against *T. gondii*; lane 3: no reaction with antibody against *T. spiralis*; lane 4: positive reaction with antibody against *H. contortus* (positive control).

The purified recombinant protein was run on 12% SDS-PAGE and the resultant band was observed at a molecular weight of 45 kDa (Figure 1A). Immunoblot analysis showed that the recombinant HcRas was recognized in sera from goats infected with *H. contortus*, while no protein was detected in the normal goat sera (Figure 1B). Furthermore, no cross-reaction was found between rHcRas and antibodies against *F. hepatica*, *T. gondii*, or *T. spiralis* (Figure 1C).

### 3.2. Serodiagnostic Potential of rHcRas

**Figure 2:**
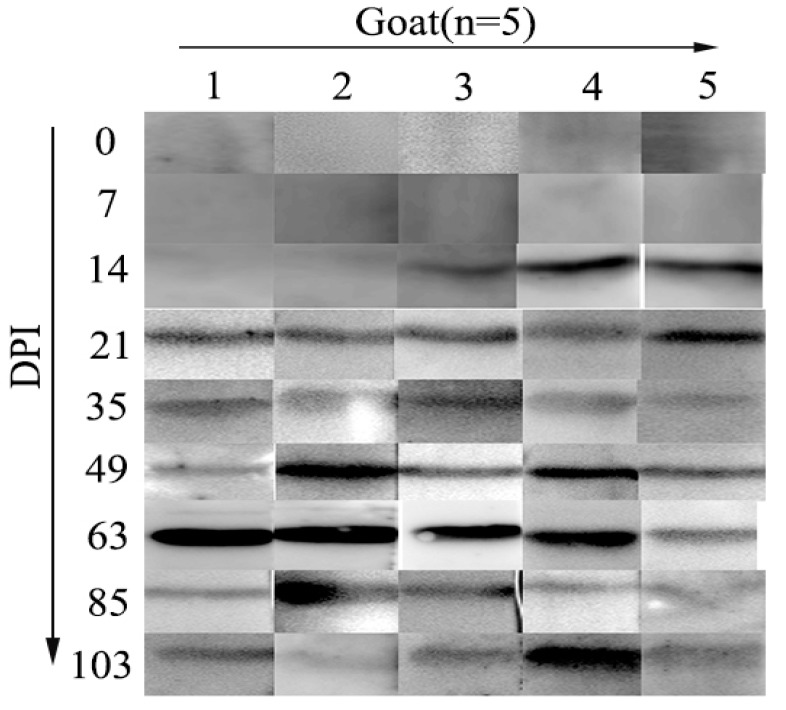
Western blotting showed the reactions between rHcRas and sera from goats (*n* = 5) infected with *H. contortus*. X axis: 1, 2, 3, 4, and 5 represent five goats. Y axis: serum collected at 0, 7, 14, 21, 35, 49, 63, 85, and 103 days post infection.

The purified antigen strongly reacted with antibodies in all of the artificially infected goats’ sera collected at 21, 35, 49, 63, 85, and 103 days post-*H. contortus*-infection days, while no IgG antibody against rHcRas was detected in the serum samples collected from goats at 0 DPI (non-infected) and 7 DPI. At 14 DPI, anti-rHcRas antibody was detected in only 3 goats (Figure 2, Table S1). 

### 3.3. Development of Indirect ELISA

**Figure 3:**
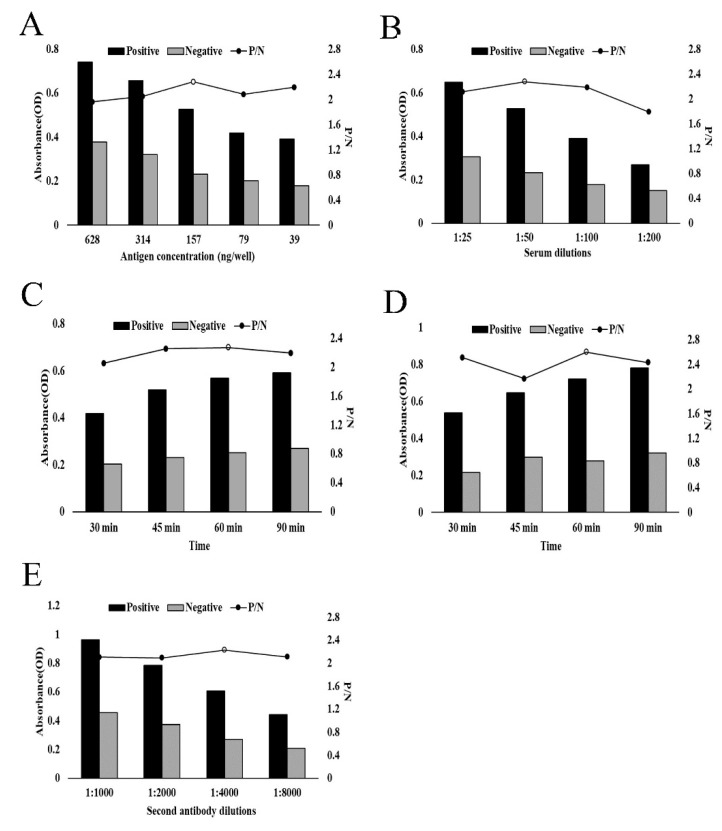
Optimization of indirect ELISA using rHcRas as the coating antigen. (**A**) Coating antigen (628, 314, 157, 79, and 39 ng of rHcRas per well). (**B**) Dilution of primary antibody (1:25, 1:50, 1:100, and 1:200). (**C**) Incubation time for primary antibody (30, 45, 60, and 90 min). (**D**) Incubation time for secondary antibody (30, 45, 60, and 90 min). (**E**) Dilution of second antibody (1:1000, 1:2000, 1:4000, and 1:8000).

The optimal dilution of the antigen (rHcRas), test sera, antibody reactivity time, and conjugate in the rHcRas ELISA were determined using a checkerboard titration. The biggest differences in the OD values for the negative and positive sera were observed when using rHcRas as the coating protein at a final concentration of 157 ng/well. The optimal dilution of the test sera was 1:50. Using these optimal rHcRas-coating protein and sera dilutions, the optimal dilution of the HRP-conjugated anti-goat IgG (second antibody) was 1:4000. Then, optimization of the other conditions was performed. The best reactive times for the serum and secondary antibody were recorded at 60 min (Figure 3). 

### 3.4. Cut-Off Value of Indirect ELISA

**Figure 4:**
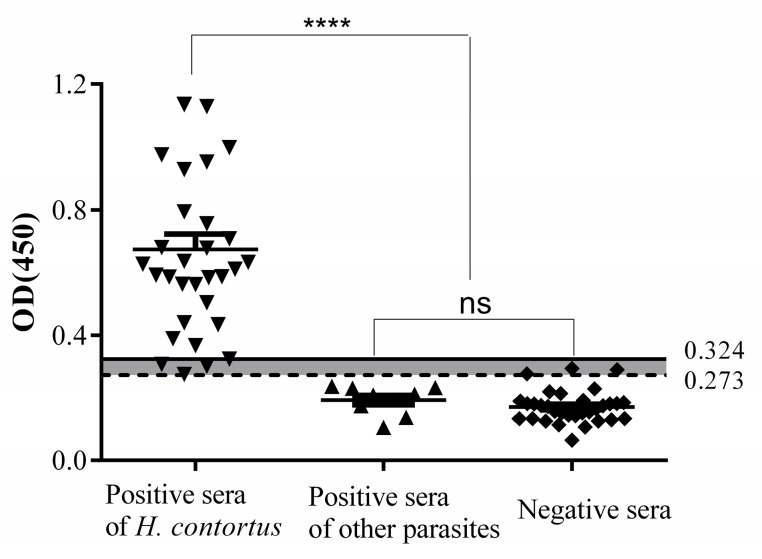
Sensitivity, specificity, and cross-reactivity of the ELISA. The solid horizontal line represents the positive cut-off value (0.324) and the dotted horizontal line represents the negative cut-off value (0.273). Four samples were observed as false negatives and three as false positives. Statistically significant differences were observed between *H. contortus*-positive sera and the other organisms’ sera (*T. gondii-, F. hepatica-*, and *T. spiralis*-positive) and *H. contortus*-negative sera. No significant differences were noted between the *H. contortus*-negative and the other parasite-positive serum samples.

In total, 32 negative and 32 positive sera were chosen for further rHcRas ELISA optimization. The positive cut-off value was determined by mean + 3SD; 0.171 + 3 × 0.051 = 0.324, while the negative cut-off value was calculated by mean + 2SD; 0.171 + 2 × 0.051 = 0.273. Therefore, the negative and positive thresholds were set at 0.273 and 0.324, respectively. Sera with absorbance values of ≥0.324 were considered positive and sera with absorbances of ≤0.273 were considered negative. The sensitivity and specificity of indirect ELISA are shown as Figure 4. The OD450 values of four positive samples and three negative samples were between the negative and positive cut off (0.273 < OD450 < 0.324); these samples were considered to be false positives and false negatives, respectively. The sensitivity of the indirect ELISA as identified by positive serum samples was 87.5% (28/32), and the specificity as identified by the negative sera was 90.6% (29/32). No cross-reaction was observed between the rHcRas and the antibodies against *T. spiralis* (*n* = 4), *F. hepatica* (*n* = 1), or *T. gondii* (*n* = 4). 

### 3.5. Determination of Repeatability

Twelve serum samples were selected randomly and compared for both intra-assay and inter-assay variations. The results showed that the intra-assay CV value of 6 samples was 4.7%–11.2% and the inter-assay CV value range was 6.5%–14.7%. The minor variation in results indicated that the new rHcRas -ELISA was reproducible (Table S2).

### 3.6. Diagnosis of H. Contortus 

**Figure 5:**
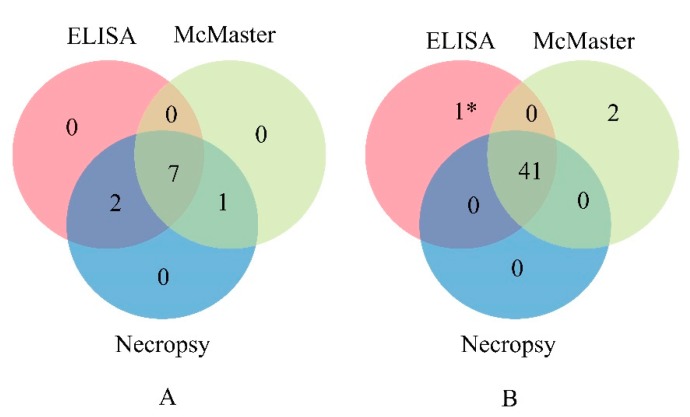
Venn diagram showing the positive (**A**) and negative (**B**) samples as tested by ELISA, McMaster, and necropsy. 1* represents that 1 sample was found to be a false positive/negative by ELISA.

Fifty-one goats were randomly selected from the field and tested using indirect ELISA, McMaster, and necropsy. The results of the indirect ELISA indicated 9 goats as positive, 41 as negative, and 1 as false assessed (negative/positive). The result of McMaster test showed 15.7% (8/51) goats were infected with *H. contortus*, which was lower than that of the indirect ELISA (17.6%, 9/51) and necropsy testing (19.6%, 10/51) (Figure 5A). Comparing the results of these three methods, 41 goats were found to be negative for *H. contortus* infection (Figure 5B). The coincidence rate between the indirect ELISA and necropsy was 98.0% ((9 + 41)/51).

## 4. Discussion

The parasitic nematode *Haemonchus contortus* is the most important parasite species of livestock. Currently, diagnosis of Haemonchosis depends upon animal health status and conventional diagnostic techniques like fecal egg counts. However, since clinical symptoms usually become apparent only when the infection is severe, these methods are time-consuming and inaccurate [5,19]. These limitations are major obstacles in the early diagnosis of this infection.

It was recently reported that concentration on the development of potential diagnostic tests was not only limited to *H. contortus* detection, but also on the development of new molecular vaccines and anthelmintic chemicals against Haemonchosis [6,20]. The antigen preparations used in diagnostic assays were primarily derived from whole worm extract, excretory and secretory worm products, sonicated egg antigens, partially purified fractions, and recombinant antigens [21]. The crude somatic antigen was evaluated for the immunodiagnosis of *H. contortus* infection [22]. However, cross-reactions between low sensitivity and different worms limited the use of these antigens. Hence, the use of purified immunochemically characterized antigens is necessary to improve the sensitivity and specificity of these tests.

Previous studies showed that Ras-Related Proteins appeared to have diverse cytobiological functions and were considered to be molecular switches because they cycle between the “on” and “off” conformations [23,24]. Previous studies reported that ESPs could be used as antigens to diagnose *H. contortus* infection in sheep [3,25]. Ras domain-containing protein (Ras) was one of the most important constituents of HcESPs (*H. contortus* excretory and secretory products) that was secreted at different larval stages of *H. contortus* [10]. These features indicated that rHcRas was potential candidate antigen for serodiagnostic tools, such as Western blotting and ELISA. In this study, indirect ELISA was performed to evaluate the potential serodiagnostic role of rHcRas protein during the prepatent period. Previously, it was reported that the antibody detection method was more sensitive than microscopic parasitological techniques and could be used in areas characterized by a low level of transmission, prevalence, and intensity [26,27]. The Western blotting analysis of this study showed that the rHcRas antigen was first detected at 14 DPI and persisted until 103 DPI. However, anti-Ras antibodies were not detected in uninfected (0 d) serum. In contrast, previous studies could not detect the infection at the early stage, rather, *H. contortus* eggs were detected in small ruminants only at 21–35 DPI [28]. Additionally, cross-reactivity of the rHcRas antigen with co-infecting pathogens (i.e., *Trichinella spiralis*, *Fasciola hepatica,* and *Toxoplasma gondii*) was not identified, which indicated high specificity. In contrast to these observations, previous studies showed that antigens of *H. contortus* revealed significant cross-reactivity with cestodes and trematodes [3], potentially due to the difference in the antigens studied. Thus, the evaluated rHcRas could act as diagnostic antigens for the detection of specific antibodies produced during the prepatent stages of *H. contortus* infection in goats.

In the current study, an indirect ELISA was developed to identify specific antibodies during the prepatent period against rHcRas, with the obtained results further confirmed by immunoblot assay. As one of the most sensitive immunoassays, ELISA offers commercial value in laboratory research and diagnostic biomarkers [29]. The combination of ELISA and WB is considered a gold standard in human medicine [30] and might also be an effective combination for goats. Determination of the lowest amount of background noise, optimization of the best antigen, and antibody dilution are crucial to improve diagnostic potential [31]. After standardization of the rHcRas-based indirect ELISA, checkerboard titration results showed the optimal antigen-coating concentration (157 ng/well), the optimal serum dilution ratio (1:50), and the optimal concentration of the rabbit anti-goat IgG (1:4000). Usually, incubation time determines the total assay time of ELISA. Incubation time of the buffer significantly affected the performance of the assay [32,33], so the incubation time for the serum and the secondary antibody was investigated. The results of this study showed an incubation time of 60 minutes for both the serum and the secondary antibody where the positive/negative value was most appropriate. 

After standardization, the ELISA-rHcRas that was developed in this investigation indicated 87.5% (28/32) sensitivity and 90.6% (29/32) specificity. The OD450 values of four positive samples and three negative samples were between the negative and positive cut-off values and were determined to be false positives and false negatives, respectively. In contrast to our ELISA results, recombinant *H. contortus* p26/23 antigen-based ELISA obtained the highest prevalence percentage (90.8%) and sensitivity (90%), however, it also revealed remarkably high false-positive numbers in healthy sheep [34]. A repeatability assessment of the indirect ELISA showed CVs of less than 15%, indicating that the indirect ELISA system was of adequate repeatable precision.

Relatively, the results of three different diagnostic assays showing an *H. contortus* prevalence of 17.6% (9/51) was recorded by the indirect ELISA, which was higher than that observed by fecal examination (15.7%, 8/51). Moreover, the presence of worms in goat abomasum confirmed the accuracy of the ELISA. However, further studies should be conducted with large sample numbers to improve these assays.

## 5. Conclusions

The indirect ELISA based on the rHcRas antigen detected anti-*H. contortus* antibodies in goat sera with improved sensitivity and specificity. In addition, the immunoblot assay developed with rHcRas was able to detect antibodies at the initial stage of infection (14 DPI) until the late stage (21–103 DPI). The results of this study concluded that rHcRas was an effective immunodiagnostic antigen to detect *H. contortus* infection during the prepatent and postpatent periods in goats. Moreover, indirect ELISA based on rHcRas also had the potential to detect *H. contortus* infection from field samples.

## Supplementary Materials

The following are available online at https://www.mdpi.com/2076-2615/10/1/138/s1, Table S1: Reactions between rHcRas and serum from goat infected with *H. contortus*; Table S2: Stability of the indirect ELISA.

## References

[1] Besier R.B., Kahn L.P., Sargison N.D., Van Wyk J.A. (2016). Diagnosis, Treatment and Management of *Haemonchus Contortus* in Small Ruminants. Adv. Parasitol..

[2] Cabardo D.E., Portugaliza H.P. (2017). Anthelmintic Activity of Moringa Oleifera Seed Aqueous and Ethanolic Extracts against *Haemonchus Contortus* Eggs and Third Stage Larvae. Int. J. Vet. Sci Med..

[3] Kandil O.M., Hendawy S.H.M., Namaky A.H., Gabrashanska M.P., Nanev V.N. (2017). Evaluation of Different *Haemonchus Contortus* Antigens for Diagnosis of Sheep Haemonchosis by Elisa and Their Cross Reactivity with Other Helminthes. J. Parasit. Dis..

[4] Ljungstrom S., Melville L., Skuce P.J., Hoglund J. (2018). Comparison of Four Diagnostic Methods for Detection and Relative Quantification of *Haemonchus Contortus* Eggs in Feces Samples. Front. Vet. Sci..

[5] Yang X., Qi M.W., Zhang Z.Z., Gao C., Wang C.Q., Lei W.Q., Tan L., Zhao J.L., Fang R., Hu M. (2017). Development and Evaluation of a Loop-Mediated Isothermal Amplification (Lamp) Assay for the Detection of *Haemonchus Contortus* in Goat Fecal Samples. J. Parasitol..

[6] Naqvi M.A., Naqvi S.Z., Memon M.A., Aimulajiang K., Haseeb M., Xu L., Song X., Li X., Yan R. (2019). Combined Use of Indirect Elisa and Western Blotting with Recombinant Hepatocellular Carcinoma-Associated Antigen 59 Is a Potential Immunodiagnostic Tool for the Detection of Prepatent *Haemonchus Contortus* Infection in Goat. Animals.

[7] Chen Y., Giri B.R., Li X., He X., Jing Z., Cheng G. (2019). Preliminary Evaluation of the Diagnostic Potential of *Schistosoma Japonicum* Extracellular Vesicle Proteins for *Schistosomiasis Japonica*. Acta Trop..

[8] Naqvi M.A., Jamil T., Naqvi S.Z., Memon M.A., Aimulajiang K., Aleem M.T., Ehsan M., Xu L., Song X., Li X. (2019). Immunodiagnostic Potential of Recombinant Tropomyosin During Prepatent *Haemonchus Contortus* Infection in Goat. Res. Vet. Sci..

[9] Aimulajiang K., Naqvi M.A.-U.-H., Chu W., Lu M., Tian X., Bu Y., Memon M.A., Li X., Xu L., Song X. (2019). Adhesion-Regulating Molecule from *Haemonchus Contortus*: Potential Antigen for Diagnosis of Early Infection in Goats. Pathogens.

[10] Gadahi J.A., Wang S., Bo G., Ehsan M., Yan R., Song X., Xu L., Li X. (2016). Proteomic Analysis of the Excretory and Secretory Proteins of *Haemonchus Contortus* (HcESP) Binding to Goat Pbmcs in Vivo Revealed Stage-Specific Binding Profiles. PLoS ONE.

[11] Bae J.W., Kim S.H., Kim D.H., Ha J.J., Yi J.K., Hwang S., Ryu B.Y., Pang M.G., Kwon W.S. (2019). Ras-Related Proteins (Rab) Are Key Proteins Related to Male Fertility Following a Unique Activation Mechanism. Reprod. Biol..

[12] Bradford M.M. (1976). A Rapid and Sensitive Method for the Quantitation of Microgram Quantities of Protein Utilizing the Principle of Protein-Dye Binding. Anal. Biochem..

[13] Akao T., Kakehi Y., Wu X.X., Kinoshita H., Takahashi T., Ogawa O., Kato T., Yoshida O. (1997). Semi-Quantitative Analysis of Telomerase Activity of Exfoliated Cells in Urine of Patients with Urothelial Cancers: Causative Factors Affecting Sensitivity and Specificity. Urol. Oncol..

[14] Debajyoti G., Bernstein J. (2019). Development of a Progesterone-Specific Ige Assay for Diagnosing Patients with Suspected Progestogen Hypersensitivity. Ann. Allergy Asthma Immunol..

[15] Deo V.K., Inagaki Y., Murhandarwati E.H., Asmara W., Miyazaki T., Kato T., Park E.Y. (2019). Sero-Diagnostic Potential of *Plasmodium Falciparum* Recombinant Merozoite Surface Protein (Msp)-3 Expressed in Silkworm. Parasitol. Int..

[16] Jansen F., Dorny P., Berkvens D., Van Hul A., Van den Broeck N., Makay C., Praet N., Gabriel S. (2016). Assessment of the Repeatability and Border-Plate Effects of the B158/B60 Enzyme-Linked-Immunosorbent Assay for the Detection of Circulating Antigens (Ag-Elisa) of *Taenia Saginata*. Vet. Parasitol..

[17] Lopes L.G., Silva M.H., Figueiredo A., Canuto K.M., Brito E.S., Ribeiro P.R.V., Souza A.S.Q., Barioni-Junior W., Esteves S.N., Chagas A.C.S. (2018). The Intake of Dry Cashew Apple Fiber Reduced Fecal Egg Counts in *Haemonchus Contortus*-Infected Sheep. Exp. Parasitol..

[18] Paswan J.K., Kumar K., Kumar A., Kumar S., Chandramoni (2016). Effect of Feeding Acacia Nilotica Pod Meal on Hematobiochemical Profile and Fecal Egg Count in Goats. Vet. World.

[19] Khan S., Zhao X., Hou Y., Yuan C., Li Y., Luo X., Liu J., Feng X. (2019). Analysis of Genome-Wide Snps Based on 2b-Rad Sequencing of Pooled Samples Reveals Signature of Selection in Different Populations of *Haemonchus Contortus*. J. Biosci..

[20] Han K., Xu L., Yan R., Song X., Li X. (2012). Molecular Cloning, Expression and Characterization of Enolase from Adult *Haemonchus Contortus*. Res. Vet. Sci..

[21] Kumar N., Das B., Jadav M.M., Solanki B.J. (2016). Immunodiagnostic Potency of Homologous Antigens for Natural *Haemonchus Contortus* Infection in Small Ruminants in Plate and Paper Enzyme Linked Immunosorbent Assay. Indian, J. Anim. Res..

[22] Gowda A.K. (2016). Sero-Prevalence of *Haemonchus Contortus* Infection in Sheep by Indirect-Elisa Using Somatic Antigen. J. Parasit. Dis..

[23] Bai X.C., Jiang Q.F., Xu M., Liang X. (2019). Rassf1 Promotes Cardiomyocyte Apoptosis after Acute Myocardial Infarction and Is Regulated by Mir-125b. J. Cell. Biochem..

[24] Munoz-Maldonado C., Zimmer Y., Medova M. (2019). A Comparative Analysis of Individual Ras Mutations in Cancer Biology. Front. Oncol..

[25] Schallig H.D., van Leeuwen M.A., Hendrikx W.M. (1994). Immune Responses of Texel Sheep to Excretory/Secretory Products of Adult *Haemonchus Contortus*. Parasitology.

[26] Xu J., Peeling R.W., Chen J.X., Wu X.H., Wu Z.D., Wang S.P., Feng T., Chen S.H., Li H., Guo J.G. (2011). Evaluation of Immunoassays for the Diagnosis of *Schistosoma Japonicum* Infection Using Archived Sera. PLoS Negl. Trop. Dis..

[27] Lv C., Hong Y., Fu Z., Lu K., Cao X., Wang T., Zhu C., Li H., Xu R., Jia B. (2016). Evaluation of Recombinant Multi-Epitope Proteins for Diagnosis of Goat Schistosomiasis by Enzyme-Linked Immunosorbent Assay. Parasit. Vectors.

[28] Schallig H.D., Hornok S., Cornelissen J.B. (1995). Comparison of Two Enzyme Immunoassays for the Detection of *Haemonchus Contortus* Infections in Sheep. Vet. Parasitol..

[29] Lin A.V. (2015). Indirect Elisa. Methods Mol. Biol..

[30] Muller N., Frei E., Nuñez S., Gottstein B. (2007). Improved Serodiagnosis of Alveolar Echinococcosis of Humans Using an in Vitro-Produced *Echinococcus Multilocularis* Antigen. J. Parasitol..

[31] Wattanaphansak S., Asawakarn T., Gebhart C.J., Deen J. (2008). Development and Validation of an Enzyme-Linked Immunosorbent Assay for the Diagnosis of Porcine Proliferative Enteropathy. J. Vet. Diagn. Invest..

[32] Kumar S., Ahirwar R., Rehman I., Nahar P. (2017). Moderate Reagent Mixing on an Orbital Shaker Reduces the Incubation Time of Enzyme-Linked Immunosorbent Assay. Anal. Biochem..

[33] Azri F.A., Sukor R., Selamat J., Abu Bakar F., Yusof N.A., Hajian R. (2018). Electrochemical Immunosensor for Detection of Aflatoxin B₁ Based on Indirect Competitive Elisa. Toxins.

[34] Kandil O.M., Gamil I.S., Hendawy S.H.M., Medhat F., El-Habit O.H. (2017). Efficacy of Glutathione-S-Transferase Purified Antigen of the Gastro-Intestinal Nematode *Haemonchus Contortus* in Diagnosis of Sheep Haemonchosis. J. Parasit. Dis..

