# Supplementary material for: Development and Potential Application of Ras Domain Containing Protein from Haemonchus contortus for Diagnosis of Goat Infection
Source: Animals (Basel). 2020 Jan 15;10(1):138. doi: 10.3390/ani10010138 (PMC7022636; doi:10.3390/ani10010138)
Supplement: Supplementary file 1 [file animals-10-00138-s001.pdf]

animals

Development and Potential Application of Ras Domain-Containing Protein from *Haemonchus contortus* for Diagnosis of Goat Infection

Kalibixiati Aimulajiang, Man Cao, Shuyi Liao, Muhammad Ali-ul-Husnain Naqvi, Xiaowei Tian, Zehua Li, Mingmin Lu, Shakeel Ahmed Lakho, Xiangrui Li, Lixin Xu, Xiaokai Song and Ruofeng Yan *

Table 1. Reactions between rHcRas and serum from goat infected with *H. contortus.*

DPI	1	2	3	4	5
0	−	−	−	−	−
7	−	−	−	−	−
14	−	−	+	+	+
21	+	+	+	+	+
35	+	+	+	+	+
49	+	+	+	+	+
63	+	+	+	+	+
85	+	+	+	+	+
103	+	+	+	+	+

Reactions between rHcRas and serum from goat (n = 5) infected with *H. contortus* were tested by WB, "+" represent positive reaction and "−" as negative.

Table 2. Repeatability of the indirect ELISA.

Intra-assay	P1	P2	P3	N1	N2	N3
R1	0.439	0.559	0.658	0.329	0.305	0.305
R2	0.466	0.469	0.62	0.277	0.27	0.283
R3	0.425	0.47	0.592	0.275	0.244	0.306
Mean	0.443	0.499	0.623	0.294	0.273	0.298
SD	0.021	0.052	0.033	0.031	0.031	0.013
**CV (%)**	**4.701**	**10.349**	**5.314**	**11.215**	**10.272**	**4.815**
**Inter-assay**	**P4**	**P5**	**P6**	**N4**	**N5**	**N6**
R-1	0.328	0.468	0.578	0.291	0.315	0.297
R-2	0.371	0.506	0.516	0.288	0.261	0.311
R-3	0.299	0.445	0.502	0.231	0.231	0.261
Mean	0.333	0.473	0.532	0.270	0.269	0.290
SD	0.036	0.031	0.040	0.034	0.043	0.026
**CV (%)**	**10.890**	**6.513**	**7.603**	**12.568**	**14.695**	**8.250**

Positive (P1, P2 and P3) and negative samples (N1, N2 and N3) were tested by indirect ELISA in the same plate by three repeats (R1, R2 and R3). Another positive (P4, P5 and P6) and negative samples (N4, N5 and N6) were tested in three different plates (R-1, R-2 and R-3). The coefficient of variation (CV) was showed as bold.
